# Associations Between the Choroidal Vascularity Index and Malignant Glaucoma After Trabeculectomy for Primary Angle Closure Glaucoma

**DOI:** 10.3389/fmed.2021.747720

**Published:** 2021-12-08

**Authors:** Chengguo Zuo, Dingqiao Wang, Xinxing Guo, Hui Xiao, Shaoyang Zheng, Mingkai Lin, Lei Fang, Xing Liu

**Affiliations:** ^1^State Key Laboratory of Ophthalmology, Zhongshan Ophthalmic Center, Sun Yat-sen University, Guangzhou, China; ^2^Wilmer Eye Institute, Johns Hopkins University, Baltimore, MD, United States

**Keywords:** choroid, choroidal vascularity index (CVI), choroidal thickness (CT), malignant glaucoma, optical coherence tomography (OCT)

## Abstract

**Purpose:** To compare the choroidal vasculature characteristics by using the choroidal vascularity index (CVI) in eyes with malignant glaucoma (MG), fellow eyes with non-MG, and eyes with uncomplicated primary angle-closure glaucoma (PACG) after trabeculectomy by spectral-domain optical coherence tomography (SD-OCT).

**Methods:** This case–control study included 53 patients diagnosed with MG after trabeculectomy. Eyes with MG (*n* = 53) and the fellow eyes with non-MG (*n* = 50) were included. Eyes with PACG without MG after trabeculectomy (*n* = 60) were also enrolled as controls. The choroidal parameters, including CVI and the subfoveal choroidal thickness (SFCT), were measured by using SD-OCT images.

**Results:** Eyes with MG and the fellow eyes showed a significantly lower CVI than eyes with PACG controls (*p* < 0.001). After adjusting for age, sex, axial length (AL), and intraocular pressure (IOP), eyes with the greater CVI [odds ratio (OR), 0.44] were significantly related to MG. The area under the receiver operating characteristic curve of the CVI was greater than that of the SFCT in the diagnosis of MG (0.911 vs. 0.840, *p* = 0.034).

**Conclusion:** Eyes with MG showed a significantly lower macular CVI than eyes with PACG controls. A higher macular CVI was an associated factor of eyes with MG. The CVI serves as a more stable and sensitive indicator for MG than the SFCT in this group of patients with PACG.

## Introduction

Malignant glaucoma (MG), also known as aqueous misdirection glaucoma or ciliary block glaucoma, was a rare complication mostly seen in penetrating ocular surgery (1). MG may result in optic nerve damage and severe vision loss (2). The pathogenesis of MG has remained elusive. However, the pathogenesis of MG is closely associated with anatomical characteristics such as a relatively large lens, a shallow anterior chamber, and an anterior rotation of the ciliary body (1).

Quigley et al. have proposed that choroidal expansion may play a role in the pathogenesis of primary angle-closure glaucoma (PACG) and even MG (3, 4). Recently, an increasing number of studies, including our previous reports, have demonstrated a thicker choroidal thickness in primary angle-closure diseases than in the non-glaucoma controls (5–7). However, many factors affect the choroidal thickness and it is relatively unstable (8). Therefore, these conclusions are still controversial. Moreover, choroidal thickness does not differentiate vascular vs. stromal changes in the choroid. Thus, choroidal thickness alone does not capture the comprehensive choroidal features.

The choroidal vascularity index (CVI), defined as the proportion of the luminal area (LA) to the total choroidal area (TCA) (9, 10), has been documented to be a more stable and reliable parameter to monitor choroidal vasculature in retinal diseases such as age-related macular degeneration, Vogt–Koyanagi–Harada disease, and in primary open-angle glaucoma (POAG) (11–16) than the subfoveal choroidal thickness (SFCT). However, no studies have investigated the choroidal vascular changes by using the CVI parameter in eyes with MG.

In this study, we evaluated the CVI in eyes with MG, fellow eyes with non-MG, and eyes with uncomplicated PACG controls to investigate the choroidal vasculature changes and compare the stability and sensitivity of the CVI with the parameter of the SFCT.

## Materials and Methods

### Participants

This retrospective study was approved by the Institutional Review Board of Zhongshan Ophthalmic Center. The study was registered at the ClinicalTrials.gov with the registration ID: NCT04220879. This study followed the statements of the Declaration of Helsinki. A total of 53 consecutive patients diagnosed with MG and a total of 60 consecutive patients diagnosed with uncomplicated PACG were included in this study. All the data of the patient were obtained from the Glaucoma Department of Zhongshan Ophthalmic Center of Sun Yat-Sen University (Guangzhou, China) between January 2010 and November 2019.

Patients were diagnosed as having MG after trabeculectomy when they met all of the following criteria: (17, 18) high or normal intraocular pressure (IOP) pretreatment, shallowing of the anterior chamber (by slit-lamp examination), patent iridotomy, absence of signs of overfiltration or bleb leakage, and absence of choroidal effusion or hemorrhage on fundus examination or B-scan ultrasonography. The average duration of MG attacked was 6 months after trabeculectomy. The inclusion criteria for eyes with MG was a diagnosis of MG following trabeculectomy for the treatment of PACG. The fellow eyes of MG patients were also included in this study. The inclusion criteria of the fellow eyes were a diagnosis of suspect primary angle-closure, primary angle-closure, or PACG, and absence of MG disease.

Three eyes without quality optical coherence tomography (OCT) images were excluded due to lens opacity. A total of 60 eyes with uncomplicated PACG from 60 patients who were followed in the same period were included as control subjects. Patient with PACG who met the criteria was enrolled to match the patient with MG recruited in the same year. If both the eyes met the inclusion criteria, the right eye was selected. The inclusion criteria of PACG controls were post-trabeculectomy and absence of MG so far. The exclusion criteria for all the subjects included an axial length (AL) <20 mm, refractive media opacity in eyes affecting the fundus observation, intraocular diseases other than glaucoma (e.g., diabetic retinopathy or non-glaucomatous optic neuropathy), and systemic diseases (hypertension, diabetes, or pituitary tumors). Eyes with a history of ocular trauma or any ocular surgery other than trabeculectomy within the past 3 months were also excluded.

The following ophthalmic evaluations were required in all the patients enrolled in this study: slit-lamp examination, IOP measured with applanation tonometry, AL measured with A- and B-scan ultrasonography (CINESCAN; Quantel Corporation Ltd., Clermont-Ferrand, France, UK), and dilated fundus examination.

### Optical Coherence Tomography Image Acquisition

**Figure 1:**
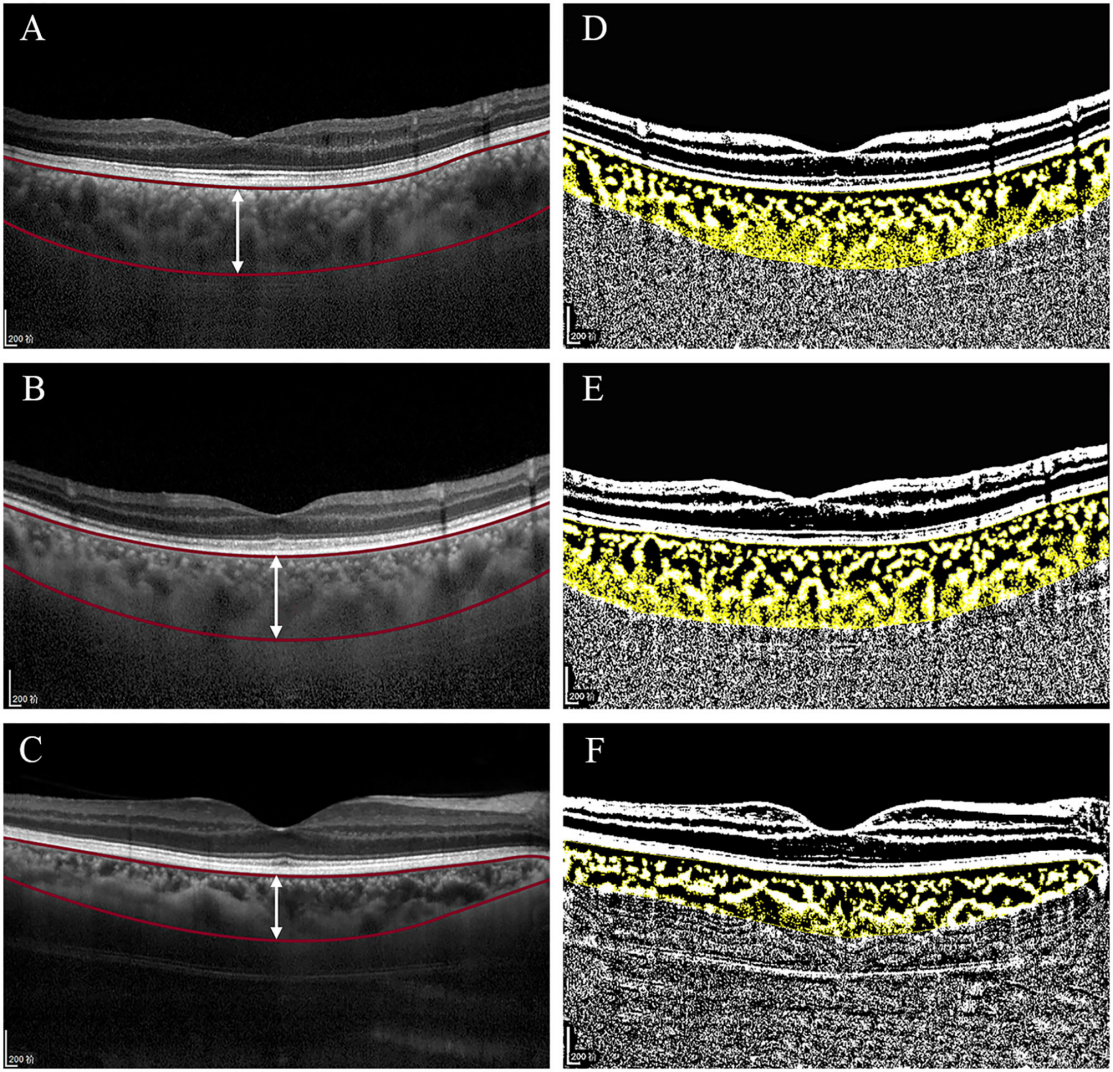
Original spectral-domain optical coherence tomography (SD-OCT) **(A-C)** and binarized SD-OCT images **(D-F)** of this study in eyes with malignant glaucoma (MG) group, the fellow eyes with non-MG group, and control eyes with uncomplicated primary angle-closure glaucoma (PACG) group. **(A-C)** Based on the prebinarized image, two horizontal red lines segment the choroid from Bruch's membrane to the choroidal–scleral interface. The vertical white line at the fovea was used for calculation of the subfoveal choroidal thickness (SFCT), which measured 485, 489, and 330 μm. **(D-F)** Based on the binarized image, the yellow block represents the total choroidal area (TCA), the choroidal vessel lumen area (LA) is represented by dark pixels, and the stromal area (SA) is represented by light pixels. The choroidal vascularity index (CVI) was 61.81, 60.56, and 68.09%.

Optical coherence tomography images with enhanced depth imaging (EDI) were obtained by using spectral-domain OCT (SD-OCT) (Spectralis HRA + OCT, Heidelberg Engineering, Heidelberg, Germany, UK) by an experienced ophthalmologist (HX). The EDI-OCT images were performed at the onset of the MG before the further surgical treatment. The scans were horizontal lines of 30° through the central fovea. The scan length was 9 mm. The quality index of each EDI image was no <22 dB. The SFCT was defined as the vertical distance from the outer surface of the retinal pigment epithelium to the inner edge of the choroid–scleral junction (Figures 1A-C). The SFCT was measured manually at the foveal center twice by one glaucoma specialist (XL) and the average value was used for analysis.

### Choroidal Vascularity Index

The details of the measurement of the CVI were described by Sonoda et al. (9). The EDI-OCT images were binarized by using the ImageJ software (ImageJ Software Inc., MD, USA) (version 1.51; http://imagej.nih.gov/ij/). The TCA was selected with the polygon tool and then converted to an 8-bit format by using Niblack's auto local threshold techniques for image binarization. Then, the image was converted into the red, green, and blue (RGB) image and the luminal and stromal regions were selected with the color threshold tool. In the binarized images (Figures 1D-F), dark pixels correspond to the LA and light pixels correspond to the stromal area (SA) (19). The CVI was measured as the ratio of the LA to the TCA. All the image assessments were performed by an experienced investigator (CZ) at two different times. Both the investigator and data collector (SZ) in this study were masked to the clinical histories of the patients.

### Data Analysis

Statistical analysis was performed by using the Statistical Package for the Social Sciences (SPSS) software version 25.0 (SPSS Incorporation, Chicago, Illinois, USA). An independent *t*-test was applied for normally distributed variables and the chi-squared test was applied for categorical variables to assess the differences in demographic and clinical characteristics between patients with the MG and controls. The Bonferroni test was used for the multiple comparisons of the choroidal parameters among eyes with MG, fellow eyes with non-MG, and eyes with PACG controls. The Pearson correlation analysis was performed to determine the associations of the CVI and the SFCT with demographics and ocular factors, respectively. The multiple logistic regression analysis was performed to determine the factors associated with MG. The receiver operating characteristic (ROC) curve analysis was performed on the choroidal parameters of the CVI and the SFCT to calculate their diagnostic power for MG. The MedCalc statistical software (MedCalc Software Ltd., Ostend, Belgium) (version 15.2.2, https://www.medcalc.org/) was used to compare the area under the ROC curves between the SFCT and the CVI. Two-sided *p* < 0.05 was considered as statistically significant. Intraobserver reliability was studied by using the Bland–Altman and the intraclass correlation coefficient (ICC) analyses.

## Results

A total of 53 MG eyes and 50 fellow non-MG eyes of 53 patients with MG and 60 eyes of 60 PACG controls were enrolled in this study.

**Figure 2:**
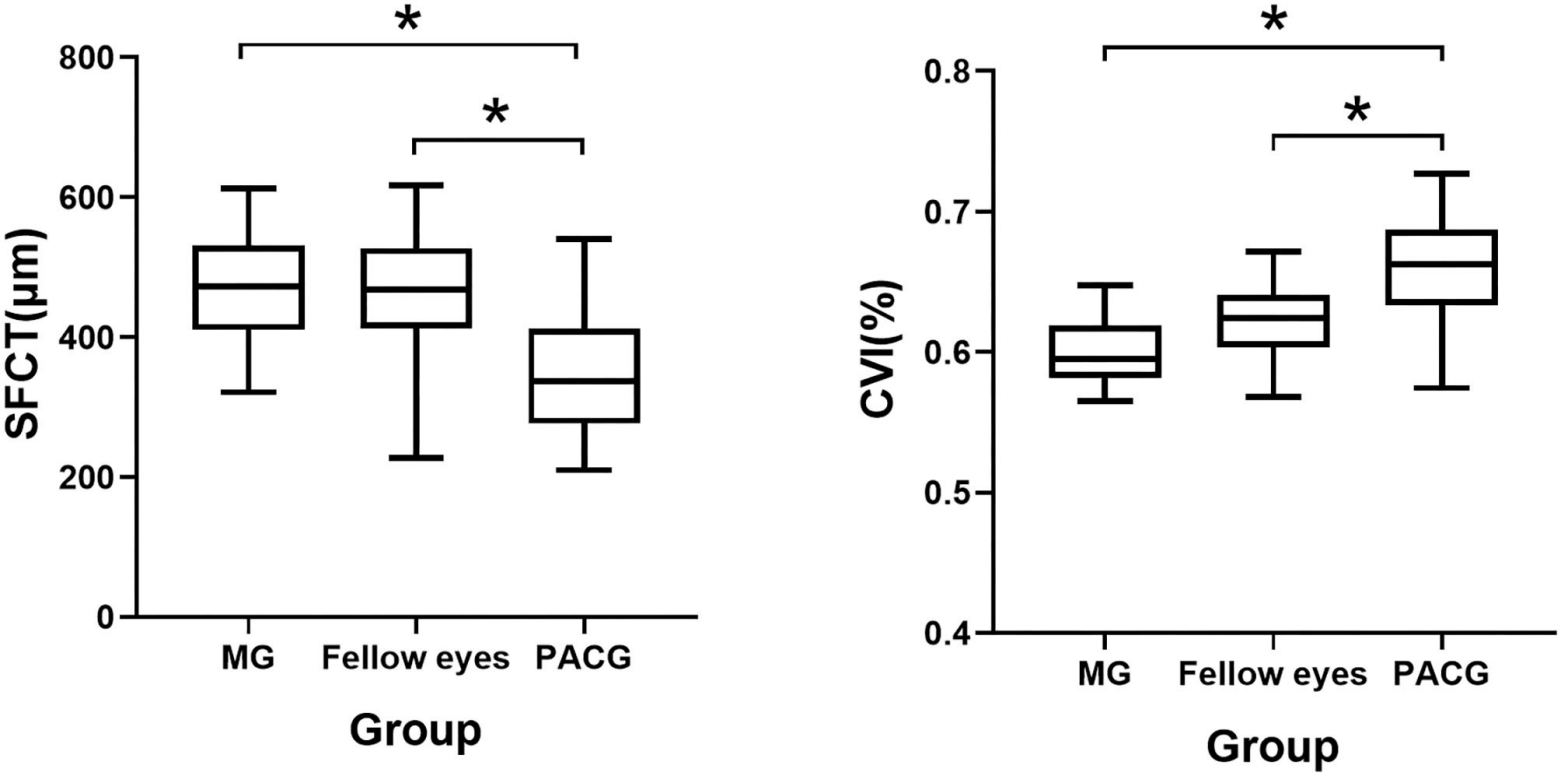
Box chart representing measurements of the CVI and the SFCT across all the groups. *p < 0.05. Error bars denote 95% CIs.

The clinical and choroidal characteristics of the patients with MG and control subjects are presented in Table 1; Figure 2. This study included 53 patients with MG with an average age of 50.3 (±11.9) years and 11 (20.8%) of the patients were male. Of the 60 PACG controls, the average age was 57.5 (±7.5) years and 34 (56.7%) of the patients were male. Patients with MG were younger and more likely to be female than the PACG controls (*p* < 0.001). Eyes with MG had a shorter AL and higher IOP than the eyes of the control group (*p* < 0.001). Fellow eyes with non-MG had a shorter AL than control eyes (*p* < 0.001). No significant differences were detected in the demographic data or choroidal parameters between eyes with MG and the fellow eyes with non-MG, except the IOP (27.1 ± 12.3 vs. 19.6 ± 9.9; *p* = 0.001).

**Table 1:** Demographic, clinical, and choroidal characteristics of eyes with MG with their fellow eyes and control eyes.

**Variables**	**MG,** ***n* = 53**	**Fellow eyes,** ***n* = 50**	**Control eyes,** ***n* = 60**	***P_**1**_***	***P_**2**_***	***P_**3**_***
Age (years)	50.3 ± 11.9	-	57.5 ± 7.5	-	**<0.001** ^†^	-
Male sex (*n*, %)	11 (20.8%)	-	34 (56.7%)	-	**<0.001** ^†^	-
Systolic blood pressure (mmHg)	123.0 ± 13.9	-	126.4 ± 16.9	-	0.252^†^	-
Diastolic blood pressure (mmHg)	77.4 ± 8.3	-	77.3 ± 8.7	-	0.958^†^	-
IOP (mmHg)	27.1 ± 12.3	19.6 ± 9.9	18.4 ± 8.1	**0.001** ^*^	**<0.001** ^*^	1^*^
Axial length (mm)	21.68 ± 1.04	21.74 ± 1.08	22.56 ± 0.73	1^*^	**<0.001** ^*^	**<0.001** ^*^
**Surgical history**
N/A	0	19	0	-	-	-
Laser iridotomy	0	31	0	-	-	-
Trabeculectomy	53	0	60	-	-	-
SFCT (μm)	471.92 ± 79.64	463.70 ± 81.10	346.05 ± 91.28	1^*^	**<0.001** ^*^	**<0.001** ^*^
LA (mm^2^)	1.35 ± 0.19	1.37 ± 0.26	1.45 ± 0.37	1^*^	0.211^*^	0.494^*^
SA (mm^2^)	0.90 ± 0.16	0.88 ± 0.26	0.77 ± 0.29	1^*^	**0.015** ^*^	0.050^*^
TCA (mm^2^)	2.25 ± 0.33	2.25 ± 0.46	2.22 ± 0.65	1^*^	1^*^	1^*^
CVI (%)	60.13 ± 2.22	61.26 ± 4.83	66.02 ± 3.58	0.314^*^	**<0.001** ^*^	**<0.001** ^*^

*Values are shown as the mean ± SD*.

*MG, malignant glaucoma; fellow eyes, fellow eyes of eyes with non-MG; IOP, intraocular pressure; SFCT, subfoveal choroidal thickness; LA, luminal area; SA, stromal area; TCA, total choroidal area; CVI, choroidal vascularity index*.

*In bold: significant at p < 0.05*.

*P_1_ eyes with MG vs. the fellow eyes with non-MG*.

*P_2_ eyes with MG vs. control eyes with PACG*.

*P_3_ fellow eyes with non-MG vs. control eyes with PACG*.

† *Student's t-test*.

* *Bonferroni correction*.

The average macular CVI was lowest in eyes with MG (60.13 ± 2.22%), lower in the fellow eyes with non-MG (61.26 ± 4.83%), and highest in control eyes with PACG (66.02 ± 3.58%). Statistically significant differences were observed in eyes with MG and the fellow eyes with non-MG compared to control eyes with PACG (*p* < 0.001, respectively). The average SFCT was thickest in eyes with MG (471.92 ± 79.64 μm), thicker in the fellow eyes with non-MG (463.70 ± 81.11 μm), and thinnest in control eyes with PACG (346.05 ± 91.28 μm). There were significant differences in the SFCT between eyes with MG and control eyes with PACG (*p* < 0.001) and between the fellow eyes with non-MG and control eyes with PACG (*p* < 0.001). Eyes with MG had a significantly larger SA than control eyes (0.90 ± 0.16 vs. 0.77 ± 0.29 mm^2^, *p* = 0.015).

The Pearson correlation analysis showed that age, AL, and systolic blood pressure were significantly negatively correlated with the SFCT (*r* = −0.486, *p* < 0.001; *r* = −0.525, *p* < 0.001; *r* = −0.259, *p* = 0.001, respectively; Table 2). However, none of these factors were significantly correlated with the CVI (*p* > 0.05 for all).

**Table 2:** The Pearson correlations between the SFCT, the CVI, and demographic factors in recruited eyes.

**Parameters**	**Factors**	**R**	***P***
SFCT	Age (years)	−0.486	**<0.001**
	sex (male)	−0.043	0.582
	Axial length (mm)	−0.525	**<0.001**
	IOP (mmHg)	−0.043	0.591
	Systolic blood pressure (mmHg)	−0.259	**0.001**
	Diastolic blood pressure (mmHg)	−0.129	0.101
CVI	Age (years)	0.133	0.077
	sex (male)	−0.028	0.715
	Axial length (mm)	0.129	0.093
	IOP (mmHg)	−0.018	0.816
	Systolic blood pressure (mmHg)	0.003	0.964
	Diastolic blood pressure (mmHg)	0.048	0.531

*R, Pearson's correlation coefficient; SFCT, subfoveal choroidal thickness; CVI, choroidal vascularity index; IOP, intraocular pressure*.

*In bold: significant at p < 0.05*.

Table 3 shows the results of the multiple logistic regression analysis of the associated factors for eyes with MG in separate models. After adjusting for age, sex, AL, and IOP, eyes with the higher CVI [odds ratio (OR), 0.44; 95% CI, 0.28–0.68] and higher LA (OR, 0.06; 95% CI, 0.01–0.57) were associated factors for MG. Eyes with the thicker SFCT (OR, 1.02; 95% CI, 1.01–1.03) were also related to MG.

**Table 3:** The multiple logistic regression analysis of the associated factors for eyes with MG.

	**MG (*****n*** **=** **53) vs. Control eyes (*****n*** **=** **60)**		
**Parameters**	**OR**	**95% CI**	***P***
SFCT (per 1 μm increase)	1.02	1.01–1.03	**<0.001**
LA (per 1 mm^2^ increase)	0.06	0.01–0.57	**0.015**
SA (per 1 mm^2^ increase)	1.78	0.21–14.90	0.596
TCA (per 1 mm^2^ increase)	0.65	0.21–2.00	0.448
CVI (per 1% increase)	0.44	0.28–0.68	**<0.001**

*OR, odds ratio; SFCT, subfoveal choroidal thickness; LA, luminal area; SA, stromal area; TCA, total choroidal area; CVI, choroidal vascularity index*.

*p: Adjusted for age, gender, axial length, and intraocular pressure (IOP) and for each factor in the table*.

*In bold: significant at p < 0.05*.

**Figure 3:**
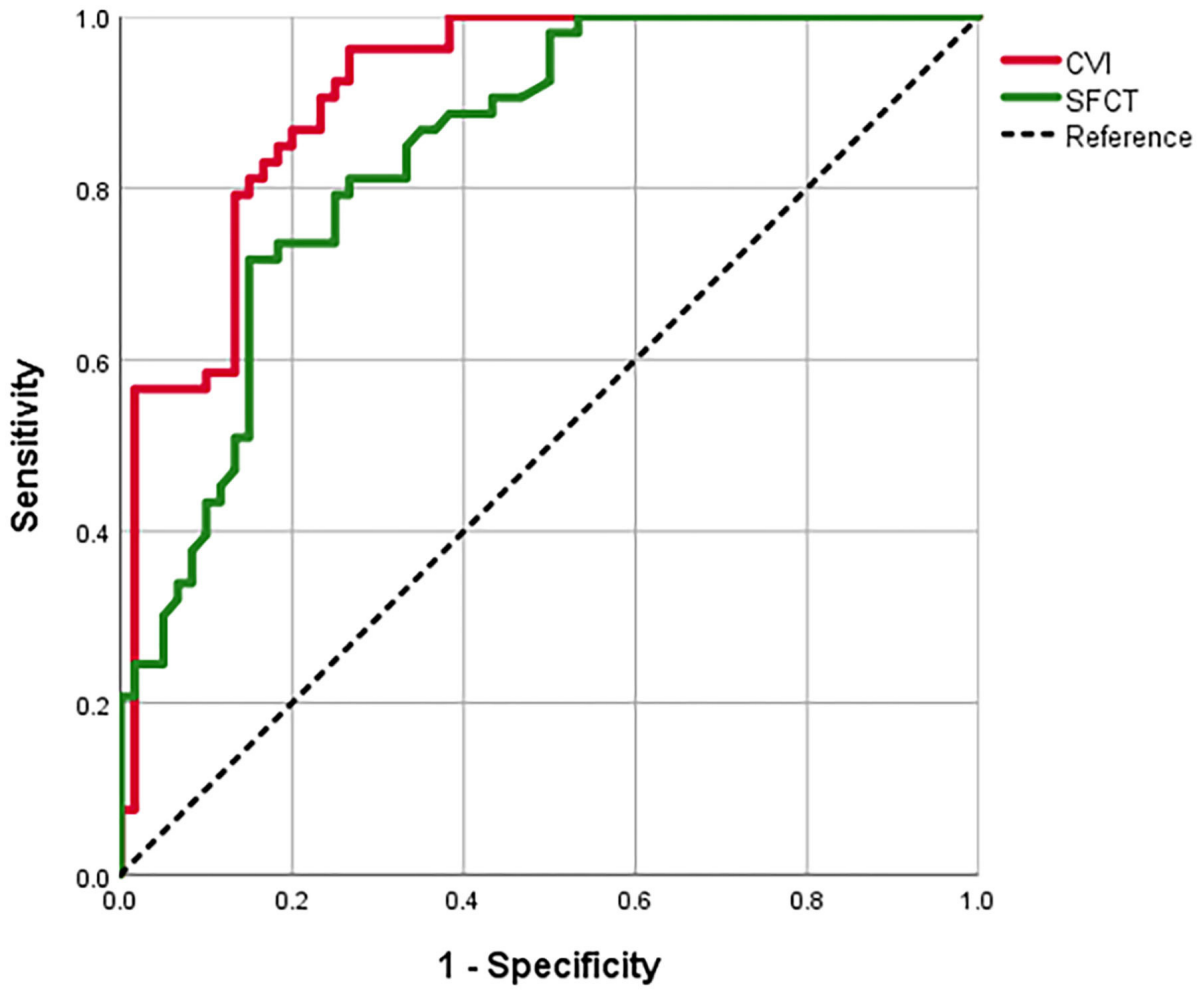
The receiver operating characteristic (ROC) curves for the SFCT and the CVI to separate eyes with MG from control eyes with POCG.

We evaluated the ROC curves for the CVI and the SFCT measures in eyes with MG vs. control eyes with uncomplicated PACG (Table 4; Figure 3). The area under the ROC curve (AUC) of the CVI was 0.911 (95% CI, 0.859–0.963; *p* < 0.001), showing a better diagnostic power than the SFCT (AUC, 0.840; 95% CI, 0.769–0.912; *p* < 0.001) in separating eyes with MG from control eyes with PACG (*p* = 0.034). The cutoff point of the CVI in the ROC curve analysis was 63.60% for the diagnosis of MG (sensitivity, 96.23%; specificity, 73.33%).

**Table 4:** Receiver operating characteristic (ROC) curves of the choroidal vascularity index (CVI) and the subfoveal choroidal thickness (SFCT) parameters to discriminate eyes with MG from control eyes with PACG.

**Parameters**	**AUC**	**95% CI**	***P***	**Cutoff point**	**Sensitivity,** **%**	**Specificity,** **%**	P^*^
CVI (%)	0.911	0.859–0.963	**<0.001**	<63.60	96.23	73.33	**0.034**
SFCT (μm)	0.840	0.769–0.912	**<0.001**	>431	71.70	85.00	

p*: Pairwise comparison of the ROC curves of the SFCT and CVI in the MedCalc software (version 15.2.2).

*Cutoff point: the maximum value at the Youden's index (sensitivity + specificity – 1)*.

*In bold: significant at p < 0.05*.

The ICC and the Bland–Altman analysis were used to assess the intraobserver reproducibility for the measurement (Table 5). The samples were selected with the random selection subroutine of the SPSS: Random Sample of Cases (version 25; IBM SPSS Corporation, Chicago, Illinois, USA). A total of 40% of the subjects were selected, which included 20 eyes with MG, 19 fellow eyes with non-MG, and 24 control eyes with uncomplicated PACG. The measurements were repeated twice at different times for reproducibility by the same investigator (CZ). The annotations were erased after grading each image after the first analysis. The ICC of the SFCT was 0.963 (*p* < 0.001) and the ICCs of the LA and TCA were 0.779 and 0.753, respectively (both *p* < 0.001). The Bland–Altman analysis showed 95% limits of agreement from −47.22 to 63.85 μm with the SFCT, from −0.35 to 0.47 mm^2^ with LA, and from −0.71 to 0.83 mm^2^ with TCA.

**Table 5:** Reproducibility of the choroidal parameters in a random subset of eyes.

**Parameters**	***N***	**Mean 1**	**Mean 2**	**ICC**	***P***	**95% LoA**
SFCT (μm)	43	405.509	397.190	0.963	**<0.001**	−47.22–63.85
LA (mm^2^)	43	1.434	1.372	0.779	**<0.001**	−0.35–0.47
TCA (mm^2^)	43	2.263	2.203	0.753	**<0.001**	−0.71–0.83

*SFCT, subfoveal choroidal thickness; LA, luminal area; TCA, total choroidal area; ICC, intraclass correlation coefficient; LoA, limits of agreement*.

*In bold: significant at p < 0.05*.

## Discussion

This study has highlighted the importance of choroidal thickness in the pathogenesis of MG (8). However, it has been widely reported that choroidal thickness can be influenced by the physiological and ocular variables (8, 20). In this study, a novel index, the CVI, was used to detect the exact changes in the choroidal vascular and stromal components in eyes with MG, the fellow eyes with non-MG, and control eyes with uncomplicated PACG (9, 10). The results showed that eyes with MG eyes and the fellow eyes with non-MG had a significantly lower macular CVI and a thicker SFCT than control eyes with uncomplicated PACG. The greater macular CVI and larger LA appeared to be associated with MG. This study also demonstrated that the CVI was more stable and sensitive than the SFCT and the parameter of the CVI was able to separate eyes with MG from control eyes with uncomplicated PACG significantly.

In this study, significant reductions in the CVI in eyes with MG and the fellow eyes with non-MG were observed compared with control eyes with uncomplicated PACG. The SA was significantly larger in eyes with MG than in control eyes with uncomplicated PACG. This study also showed a significantly thicker SFCT in both the eyes of the patients with MG than in control eyes with uncomplicated PACG. However, there were no significant differences in the CVI, TCA, LA, SA and the SFCT between eyes with MG and the fellow eyes with non-MG. The results indicated that the fellow eyes with non-MG may have a similar anatomic structure of the choroid as eyes with MG, representing a potentially high risk of MG disease (7, 17).

To compare the stability of the CVI and the SFCT, we evaluated factors that may be correlated with the SFCT and the CVI by the Pearson correlation analysis. Our results showed that the SFCT was significantly associated with age, AL, and systolic blood pressure. However, none of these three factors were statistically significantly correlated with the CVI. Our results were consistent with previous studies that observed that the SFCT may be susceptible to the physiological and ocular factors in other diseases (8, 20). Furthermore, several studies have proposed that the SFCT shows variability due to factors such as age, refraction, and AL (21, 22). It has been proposed that the CVI has less variability and is more reliable in diseases such as primary open-angle glaucoma (POAG), polypoidal choroidal vasculopathy, and panuveitis (11, 23–25) than the SFCT. In general, similar to the research of Liu et al. and Adhi et al., our results indicated that the CVI is a more stable surrogate parameter for MG than the SFCT (23, 26). Furthermore, we also investigated the reproducibility of the choroidal parameters. The results revealed a good reproducibility of the SFCT, the LA, and the TCA measurements.

After adjusting for age, sex, AL, and IOP, a greater CVI and greater LA were significantly independently associated with eyes with MG. The CVI was calculated from the binarized images from SD-OCT scans and was described by the ratio of the LA to the TCA. Thus, we speculated that a lower LA was a main contributing factor for a decline in the CVI. The reduction in the LA could be explained by a reduction in the choroidal blood supply. To the best of our knowledge, there was no investigation examining choroidal vascular characteristics in the patients with MG. Park and Cho found a significantly lower CVI and the LA in POAG eyes than healthy eyes (11). They speculated that choroidal ischemia may be related to glaucomatous eyes. Lee et al. and Kim et al. have observed a choroidal microvasculature dropout in POAG eyes on OCT angiography and indocyanine green angiography compared to normal controls (27, 28). Taken together, our findings demonstrated possible choroidal ischemia in patients with MG. Although the difference did not reach statistical significance, we found that eyes with MG had a larger SA than control eyes with uncomplicated PACG. We speculated that the relative increase in the SA may be closely associated with inflammatory factors in the choroid. It has been reported that inflammation may be involved in the pathology of glaucoma (29) and inflammatory cells can target the choroidal stroma, resulting in stromal edema and a lower CVI (30, 31). Therefore, the significant decrease in the LA and the relatively increased SA collectively led to a reduction in the CVI. We speculated that uveal effusion might happen in eyes with MG and lead to the choroidal expansions (4, 32, 33). Consistent with our hypothesis, Sakai et al. proposed that uveal effusion may be associated with eyes with MG and unaffected fellow eyes of PACG or chronic primary angle-closure eyes (34). The uveal effusion might be caused by the inflammation after surgery, which might lead to the destruction of blood–choroid barrier and the exudation of protein in the extravascular choroid. The osmotic force in the choroidal vessels was reduced (4). This could lead to the lower LA, the relatively larger SA, and the lower CVI in eyes with MG. However, uveal effusion in the fellow eyes with MG might be idiopathic. At present, many studies including us cannot explain the reason for the choroidal characteristics of the fellow eyes (34, 35).

Additionally, in this study, a thicker SFCT was found to be an associated factor for eyes with MG after adjusting for age, sex, AL, and IOP. The result of a thicker SFCT was compatible with the findings of our previous study that the choroidal thickness in eyes with MG increased during the onset of MG compared with control eyes with uncomplicated PACG (7). Similarly, Quigley et al. and Zhang et al. provided an assumption that choroidal expansion could play a significant role in the pathogenesis of PACG and even MG, which probably increases pressure in each compartment of the eye, causing a shallow anterior chamber (3, 4, 36). Eyes with MG and the fellow eyes with non-MG had significantly shorter AL values than control eyes with uncomplicated PACG. We also found that MG is more common in younger females among the patients with PACG. These findings were consistent with our previous study and other studies (7, 37).

To compare the sensitivity of the CVI and the SFCT for the diagnosis of MG, the ROC curve analysis was used. In separating eyes with MG from control eyes with uncomplicated PACG, we observed that the CVI displayed a superior diagnostic sensitivity compared to the SFCT. It suggests that the CVI indicator has significantly higher diagnostic potential for MG than the SFCT. Furthermore, the CVI was able to separate eyes with MG from control eyes with uncomplicated PACG significantly and the cutoff point in the ROC curve was 63.60% (sensitivity, 96.23%; specificity, 73.33%). We speculated that a CVI larger than 63.60% may indicate a decreased risk of MG, while a CVI lower than 63.60% may indicate an increased risk of MG. Our results indicated that the CVI could be introduced as a relatively more sensitive parameter for monitoring the choroidal status of MG.

There were some limitations in this study. The sample size of this study was relatively small and may not be representative of other cases with MG or PACG. This was a cross-sectional, observational study and we could not obtain the OCT images of the patients with MG after their treatment of operation. The OCT images in the onset of MG were measured, while images before the onset of MG were not obtained for analysis. The OCT images were only measured once, so it might affect the overall analysis of the pathogenesis of MG (38). Besides, the CVI may not represent the entire status of the global choroidal structure due to its two-dimensional nature. Therefore, the application of the CVI in MG needs to be validated in a prospective and longitudinal clinical trial with a larger sample size.

In conclusion, the macular CVI was significantly lower in both the eyes with MG and the fellow eyes with non-MG than control eyes with uncomplicated PACG. Larger macular CVI and LA were the associated factors for MG. The CVI might be a more stable and sensitive parameter than the SFCT for choroidal evaluation in MG. The CVI is likely a promising indicator for evaluating choroidal vascular changes in patients with MG.

## Data Availability Statement

The original contributions presented in the study are included in the article/supplementary material, further inquiries can be directed to the corresponding author/s.

## Ethics Statement

The studies involving human participants were reviewed and approved by the Institutional Review Board of Zhongshan Ophthalmic Center. The study was registered at ClinicalTrials.gov with the registration ID: NCT04220879.

## Author Contributions

XL and CZ contributed to the design and conduct of the study. DW and HX contributed to the collection of the data. DW and SZ contributed to the analysis of the data. DW and CZ contributed to the preparation of the manuscript. XL, ML, CZ, XG, and LF contributed to the review and final approval of the manuscript. All authors contributed to the article and approved the submitted version.

## Funding

This study was supported by the National Natural Science Foundation of China (Grant 81970808), the Guangdong Natural Science Foundation (Grant 2020A1515010121), and the Fundamental Research Funds of the State Key Laboratory of Ophthalmology.

## Conflict of Interest

The authors declare that the research was conducted in the absence of any commercial or financial relationships that could be construed as a potential conflict of interest.

## Publisher's Note

All claims expressed in this article are solely those of the authors and do not necessarily represent those of their affiliated organizations, or those of the publisher, the editors and the reviewers. Any product that may be evaluated in this article, or claim that may be made by its manufacturer, is not guaranteed or endorsed by the publisher.
